# Evaluation of Pregnant Women’s Knowledge About Preeclampsia in the Kurdistan Region of Iraq: A Cross-Sectional Study

**DOI:** 10.7759/cureus.64134

**Published:** 2024-07-09

**Authors:** Paywand S Fadhil, Maryam B Mahmmod

**Affiliations:** 1 Department of Maternity and Neonate Nursing, College of Nursing, University of Sulaimani, Sulaimani, IRQ; 2 Department of Midwifery, Erbil Medical Technical Institute, Erbil Polytechnic University, Erbil, IRQ; 3 Department of Medical Education, College of Medicine, University of Sulaimani, Sulaimani, IRQ

**Keywords:** knowledge, iraq, kurdistan region, pregnant women, mothers' knowledge, preeclampsia

## Abstract

Introduction: Iraqi pregnant women have a higher prevalence of preeclampsia (PE) compared to pregnant women in neighboring developing nations. Several maternal characteristics, such as gestational weight gain (GWG), mode of delivery, and neonatal complications, have been linked to PE. The aim of this study is to evaluate the level of knowledge of pregnant women about PE.

Methods: This cross-sectional study was conducted in the Kurdistan region in Iraq from 2022 to 2023. Data on women's knowledge was collected using a structured questionnaire consisting of 12 questions. The collected data was then analyzed using statistical methods such as the Mann-Whitney U test, Kruskal-Wallis test, and linear regression.

Results: A total of 200 pregnant women diagnosed with PE and hypertension were included in the present study. The majority of participants (n=85; 42.5%) were between the ages of 28 and 37. Additionally, most participants (n=129; 64.5%) were from urban areas, with 45% (n=90) of them being obese. As for the participants' knowledge about PE, only 24.55% (n=49) were unaware of the correct answer, while 61% (n=122) stated that family history is not a risk factor for PE. On the other hand, 60% (n=120) of the participants were aware that previous PE is a risk factor for the current pregnancy, and 58.5% (n=117) indicated the importance of urine tests for pregnant women. Overall, the majority (n=144; 72%) had a low level of knowledge, while a small proportion (n=21; 10.5%) had good and high knowledge about PE.

Conclusion: The present study provides a comprehensive assessment of the knowledge of PE among Kurdish women, highlighting specific areas where intervention and education could potentially yield significant impact.

## Introduction

Maternal mortality is primarily caused by preeclampsia (PE), a condition that affects nearly one in 10 births worldwide [1,2]. PE is the second leading cause of direct maternal deaths, resulting in approximately 70,000 fatalities annually [3]. However, the impact of PE-related morbidity and mortality in low-income countries is still uncertain. PE, being one of the top five causes of maternal mortality, accounts for 16% of all direct maternal deaths [4,5].

Iraqi pregnant women have a higher prevalence of PE compared to pregnant women in other neighboring developing countries. Various maternal characteristics, such as gestational weight gain (GWG) and surgical delivery, as well as neonatal issues, play a role in PE [6].

In their 2012 study, Altaei and Mohammad emphasized the importance of considering crucial risk factors when assessing and diagnosing PE [7]. These factors include gestational age, family history, multifetal pregnancy, previous abortion, medical history (e.g., diabetes and urinary tract infections), and social status [7]. Pregnant women with PE exhibit significantly lower levels of vascular endothelial growth factor (VEGF) compared to those with normal blood pressure, indicating severe endothelial dysfunction. Consequently, widespread vasoconstriction occurs, leading to proteinuria and hypertension [8]. Implementing evidence-based interventions to enhance pregnant women's knowledge and attitudes toward PE could effectively prevent most PE-related deaths [9]. Enhancing community awareness and changing attitudes towards hypertension during pregnancy, as well as its complications such as eclampsia, can effectively address the problem of delayed recognition and treatment of the disease. As a result, implementing such measures can significantly reduce maternal and perinatal morbidity and mortality rates that are associated with these delays [9]. However, research has shown that pregnant women often have insufficient understanding and misconceptions about PE [5,10]. A study conducted at a Tanzanian district hospital revealed that 60% of the participants were unaware of the potential risks associated with PE [11]. Ignorance has been identified as a risk factor for engaging in behaviors that increase the likelihood of developing PE. Furthermore, inadequate comprehension of the condition can cause anxiety and stress within the family [11,12].

PE and eclampsia were found to be associated with hypertension during pregnancy in 25.1% of cases, 9.8%, and 1.4% of cases, respectively [13]. Approximately 36% of maternal mortality cases were attributed to hypertension, with eclampsia emerging as the predominant underlying cause [13].

The Sustainable Development Goals (SDGs) require a greater level of commitment from countries compared to the Millennium Development Goals (MDGs), thus necessitating diligent efforts to achieve them [14]. Pregnant women's negative attitudes and limited understanding contribute to the morbidity and mortality associated with PE [15]. However, diagnosing and preventing PE pose significant challenges, including difficulties in diagnosis and management, delays in transportation, inadequate healthcare infrastructure worldwide, and a shortage of qualified medical professionals [10].

Transitioning from clinic-based care to community-based case identification and treatment is a crucial intervention strategy, particularly in resource-constrained areas, to address the burden of PE-related outcomes. Research conducted worldwide has demonstrated significant variations in pregnant women's knowledge, attitudes, and perceptions regarding PE, which can directly or indirectly influence their healthcare-seeking behavior and contribute to maternal mortality and morbidity [11]. Therefore, it is necessary to assess pregnant women's baseline knowledge about PE, particularly among high-risk groups.

Previous studies conducted in the United States and a few African countries have indicated generally poor levels of PE knowledge among women [5,11,16]. However, no research has been conducted to evaluate the level of PE knowledge in the Kurdistan region of Iraq. Thus, the objective of this study is to assess pregnant women's knowledge about PE in this geographical area.

## Materials and methods

Study design and setting

This cross-sectional study was conducted at the antenatal care unit of Rizgary Teaching Hospital in Erbil, which is located in the Kurdistan region of Iraq. The study was conducted between 2022 and 2023. Rizgary Teaching Hospital is associated with Hawler Medical University, Erbil, and serves a large population from the university and the surrounding community. The hospital's public health department provides prenatal and postnatal care for childbirth and mortality cases.

Participants and sample size calculation

The sample size for this investigation was determined using Epi Info, Version 7.2.5 (Centers for Disease Control and Prevention, Atlanta, GA). Based on the estimated prevalence of PE in Iraq, which was 4.79% [6] at a 95% confidence level, a minimum sample size of 186 was required. This calculation took into account a response distribution of 50, a margin of error of 5%, a study power of 80%, and a design effect of 1. However, to increase the statistical power, we enrolled 200 consecutive consenting pregnant women in the trial. All expectant mothers who provided their informed consent and met the study's eligibility criteria were eligible to participate, except those who were pregnant without PE and in critical condition.

Study tools and measures

Data was collected from all enrolled individuals using a standardized and well-structured questionnaire administered by the investigator. The questionnaire (Appendix A) was developed based on previous research with similar objectives [5,11,16,17] and was validated by public health specialists to ensure its reliability. If necessary, the questionnaire was translated into the participants' native languages.

The questionnaire gathered sociodemographic information and collected data on the history of PE, including details about the diagnosis of PE, age, educational level, occupation, economic status, place of residence, and BMI. Additional sections of the questionnaire covered previous pregnancies and maternal background, including gravida, parity, and history of fetal loss. Clinical and laboratory measurements, such as systolic and diastolic blood pressure, hemoglobin levels, and albumin levels, were also recorded (Appendix B). To assess participants' knowledge of PE, a series of questions were included that focused on awareness, signs and symptoms, risk factors, and complications associated with the condition. These questions were presented in a closed-ended format with predetermined choices. For example, participants were asked whether they knew the definition of PE, and they could select from response options indicating varying levels of understanding. Participants' knowledge levels were evaluated using a point system, where correct answers were awarded a score of one (1), and incorrect or unanswered questions received a score of zero (0). The overall knowledge score was reported as a percentage, and participants' knowledge levels were categorized into three tiers using Bloom's cut-off points: low (<4.1), moderate (4.1-8), and high (8.1-12) [15,18].

Pilot study

To assess the reliability of the questionnaire employed in this preliminary study, Cronbach's alpha coefficient was calculated for the PE knowledge scale. The internal consistency of the questionnaire scale was evaluated using data collected from a sample of 30 participants who participated in the pilot investigation. The resulting Cronbach's alpha coefficient for the PE knowledge scales was found to be 0.75, indicating a satisfactory level of internal consistency among the items included in the questionnaire.

Ethical approval and informed consent

This study was conducted in accordance with the ethical principles outlined in the Declaration of Helsinki. Ethical approval was obtained from the Ethics Committee of the College of Nursing at the University of Sulaimani, identified as ID 127 on October 30, 2022. Before providing written informed consent, participants were informed about the purpose, procedures, potential risks, and benefits of the study. Throughout the study, participant data confidentiality was strictly maintained. Participation in the study was voluntary, and participants had the right to withdraw at any time without facing any penalty. Measures were taken to minimize potential harm and ensure the well-being of the participants. To protect participant identities, the data were anonymized and aggregated for analysis.

Statistical analysis

To determine the normality of the data, the Kolmogorov-Smirnov test was employed. The results of this test indicate that our data is not normally distributed. As a result, non-parametric tests were conducted. Descriptive statistics were used to describe the sociodemographic characteristics of the participants. The Mann-Whitney U test and Kruskal test were then used to identify any associations. Afterward, a linear regression analysis was conducted to analyze all factors that showed statistical significance. A p-value of 0.05 or less was considered to be statistically significant. The statistical software packages IBM SPSS Statistics for Windows, Version 27 (IBM Corp., Armonk, NY), and GraphPad Prism, Version 8.0.2 (GraphPad Software, Inc., San Diego, CA) were used for data analysis.

## Results

A total of 200 pregnant women diagnosed with PE and hypertension were included in the current study. The majority of them had pregnancy-induced hypertension (PIH) (n=122, 61%). Additionally, women aged 28-37 years constituted the largest group of participants, accounting for 42.5% (n=85) of the sample. In terms of educational level, 56.5% (n=113) of the participants had completed primary education. Moreover, the majority of the participants did not have an occupation (n=185; 92.5%). Concerning economic status, 38% (n=76) of the participants reported having sufficient income for their livelihood. Furthermore, the majority of the sample (n=129; 64.5%) resided in urban areas, and a significant proportion of them (n=90; 45%) were obese (Table 1).

**Table 1:** Socio-demographic characteristics of the participants. BMI = Body mass index. The data has been represented as N and %.

Variables	Frequency (percentage)
Presence of the disease	
Preeclampsia	78 (39)
Pregnancy-induced hypertension (PIH)	122 (61)
Maternal age (year)	
18-27	78 (39)
28-37	85 (42.5)
38-47	37 (18.5)
Educational level	
Illiterate	30 (15)
Primary	113 (56.5)
Secondary	38 (19)
Institute and university	19 (9.5)
Occupation	
Employed	15 (7.5)
Non-employed	185 (92.5)
Economic status	
Low	69 (34.5)
Enough for life	76 (38)
Good	55 (27.5)
Resident place	
Urban	129 (64.5)
Rural	54 (27)
Sub-urban	17 (8.5)
BMI	
Normal	3 (1.5)
Overweight	107 (35.5)
Obese	90 (45)

Table 2 provides a summary of the participants' reproductive histories. In terms of gravida status, the majority of participants were multigravida (two to five pregnancies), totaling 143 women (71.5%). It is important to note that there were fewer primigravida women (first pregnancy), with only 42 participants (2%). Grand multigravida women (≥6 pregnancies) accounted for 15 participants (7.5%). Parity data revealed that multipara women (two to five births) were the most prevalent, representing 146 participants (73%). Nulliparous women (no births) made up 39 participants (19.5%), while primiparous women (one birth) were the least common, with only three participants (1.5%). Grand multipara women (≥6 births) numbered 12 (6%). In terms of abortion history, 103 participants (51.5%) had never experienced an abortion. A significant proportion, 89 participants (44.5%), had undergone one to two abortions, while only eight participants (4%) had a history of three to four abortions. A minority of participants reported the presence of deceased infants. The majority, 176 women (88%), reported no history of infant death, whereas 24 participants (12%) had experienced the death of one infant (Table 2).

**Table 2:** Maternal history of the pregnant women. The data has been represented as N and %.

Variable	Frequency (percentage)
Gravida	
Primigravida	42 (2)
Multigravida (2-5)	143 (71.5)
Gran multigravida (≥6)	15 (7.5)
Parity	
Nullipara	39 (19.5)
Primipara (1)	3 (1.5)
Multipara (2-5)	146 (73)
Grand multipara (≥6)	12 (6)
Abortion	
None	103 (51.5)
1-2 times	89(44.5)
3-4 times	8 (4)
Presence of dead infant	
None	176 (88)
One	24 (12)

Table 3 presents a range of clinical parameters among the participants. It is widely recognized that a systolic blood pressure greater than 130 mm/Hg indicates hypertension, whereas a diastolic blood pressure greater than 80 mm/Hg indicates hypotension. The results show that an overwhelming majority of 192 participants (n=192; 96%) were diagnosed with hypertension based on their systolic blood pressure. A mere five participants (2.5%) experienced hypotension, while only three participants (1.5%) had normal systolic blood pressure levels. The distribution of diastolic blood pressure was more varied. A majority of 114 participants (57%) had normal diastolic blood pressure, whereas 72 participants (36%) were diagnosed with hypertension. Hypotension was observed in 14 participants (7%). Hemoglobin (Hb) levels indicated a high prevalence of anemia, with 188 participants (94%) exhibiting anemia. Conversely, only 12 participants (6%) had normal Hb levels. Albumin levels demonstrated that most participants had normal levels, with 175 participants (87.5%) falling within this category. Low albumin levels were observed in 16 participants (8%), while high albumin levels were found in nine participants (4.5%) (Table 3).

**Table 3:** Clinical and laboratory investigations. BP = Blood pressure, Hb  = Hemoglobin. The data has been represented as N and %.

Variable	Frequency (percentage)
Systolic blood pressure	
Hypotension	5 (2.5)
Normal BP	3 (1.5)
Hypertension	192 (96)
Diastolic blood pressure	
Hypotension	14 (7)
Normal BP	114 (57)
Hypertension	72 (36)
Hb level	
Anaemia	188 (94)
Normal Hb	12 (6)
Albumin level	
Low	16 (8)
Normal	175 (87.5)
High	9 (4.5)

The responses regarding knowledge and awareness of PE are presented concisely in Table 4. The majority of respondents correctly identified that PIH and PE present as hypertension after 20 weeks of gestation, with 151 respondents (75.5%) answering correctly, while 49 respondents (24.5%) provided incorrect answers. Regarding risk factors, 122 participants (61%) accurately recognized family history of hypertension as a risk factor for hypertensive disorders in pregnancy (HDP)/PE, while 78 participants (39%) did not.

Knowledge about signs and symptoms of PE was well understood by 133 participants (66.5%), while 67 participants (33.5%) did not have accurate knowledge. Participants showed less awareness of certain preventive measures and outcomes associated with HDP and PE. For example, only 64 participants (32%) correctly identified pre-pregnancy obesity as a risk factor, compared to 136 participants (68%) who did not.

The importance of monitoring health indicators during pregnancy was also assessed. The majority, 137 participants (68.5%), correctly acknowledged the need to measure blood pressure, while 63 participants (31.5%) did not. Similarly, 117 participants (58.5%) recognized the importance of urine tests, while 83 participants (41.5%) did not. In terms of treatment and consequences, opinions were more evenly divided. Approximately half of the participants believed that diet (101 participants, 50.5%) and exercise (117 participants, 58.5%) could treat PIH and PE, while the other half did not share this belief. Finally, awareness of the potential harm if PIH and PE are left untreated was high, with 177 participants (88.5%) acknowledging the risk, while 23 participants (11.5%) did not (Table 4).

**Table 4:** Knowledge of the pregnant women regarding preeclampsia. PIH = Pregnancy-induced hypertension, HDP = Hypertensive disorders of pregnancy. The data has been represented as N and %.

Questions	Incorrect n (%)	Correct n (%)
PIH and preeclampsia become hypertension after 20 weeks?	49 (24.5)	151 (75.5)
Is a family history of hypertension a risk factor for HDP/preeclampsia?	122 (61)	78 (39)
Do you know the signs and symptoms of preeclampsia?	67 (33.5)	133 (66.5)
Is pre-pregnancy obesity a risk factor for PIH and preeclampsia?	136 (68)	64 (32)
Are PIH and preeclampsia in previous pregnancy a risk factor for PIH and preeclampsia in current pregnancy?	80 (40)	120 (60)
Is rapid weight gain in pregnancy a risk factor for HDP/preeclampsia in pregnancy?	117 (58.5)	83 (41.5)
Is measuring urine tests necessary in pregnancy?	83 (41.5)	117 (58.5)
Is measuring blood pressure necessary in pregnancy?	137 (68.5)	63 (31.5)
Can diet treat PIH and preeclampsia?	99 (49.5)	101 (50.5)
Can exercise treat PIH and preeclampsia?	83 (41.5)	117 (58.5)
Can a baby be harmed if PIH and preeclampsia are not treated?	177 (88.5)	23 (11.5)
Are mothers with PIH and preeclampsia at risk of developing chronic hypertension in the future?	170 (85)	30 (15)

**Figure 1:**
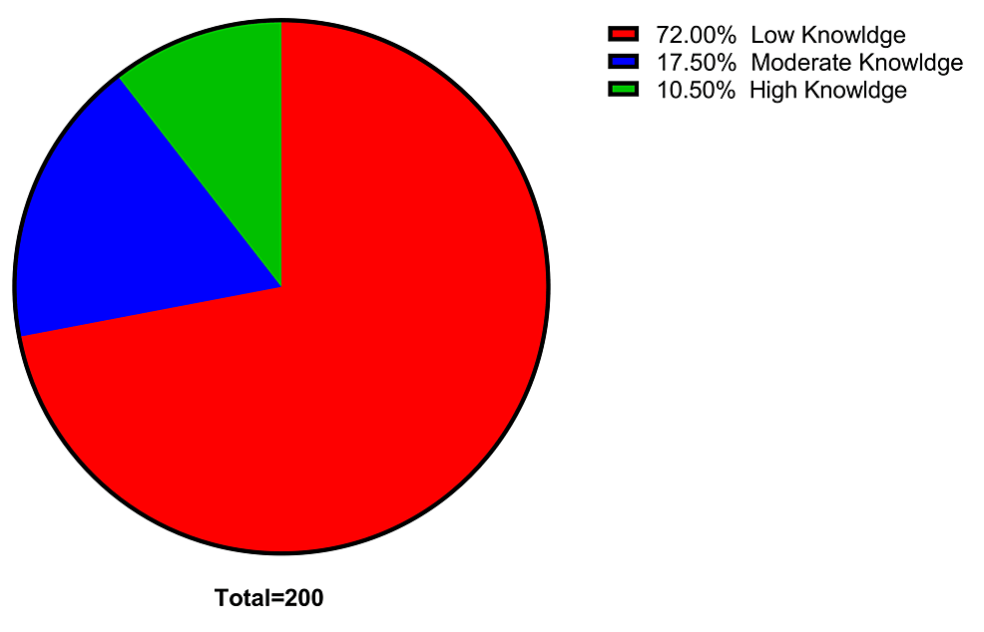
Levels of knowledge toward PE. PE = Preeclampsia. The data has been represented as %.

Overall, the majority of participants (72%) had a low level of knowledge regarding PE, while only a small percentage (10.5%) showed a high level of knowledge (Figure 1).

**Figure 2:**
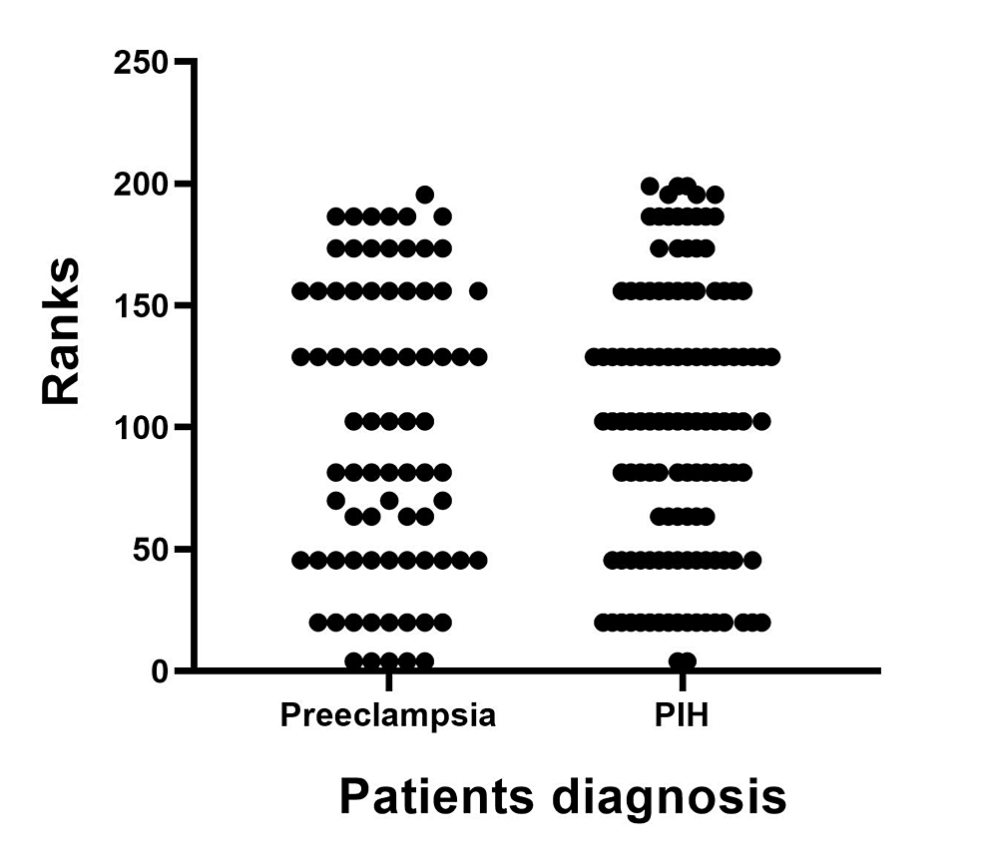
Median score between PIH and PE patients. PIH = Pregnancy-induced hypertension, PE = Preeclampsia. The Mann-Whitney U test was used. p≤0.05

**Figure 3:**
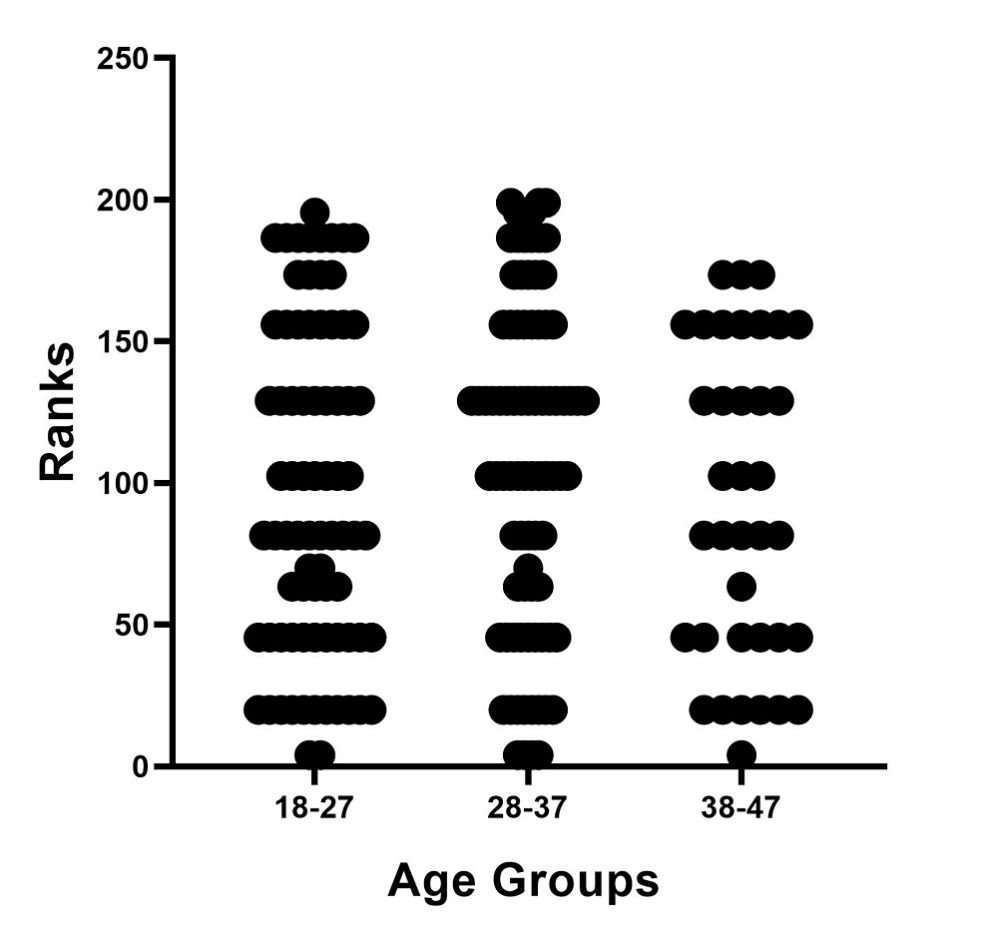
Difference among age groups regarding knowledge score. The Mann-Whitney U test was used. p≤0.05

**Figure 4:**
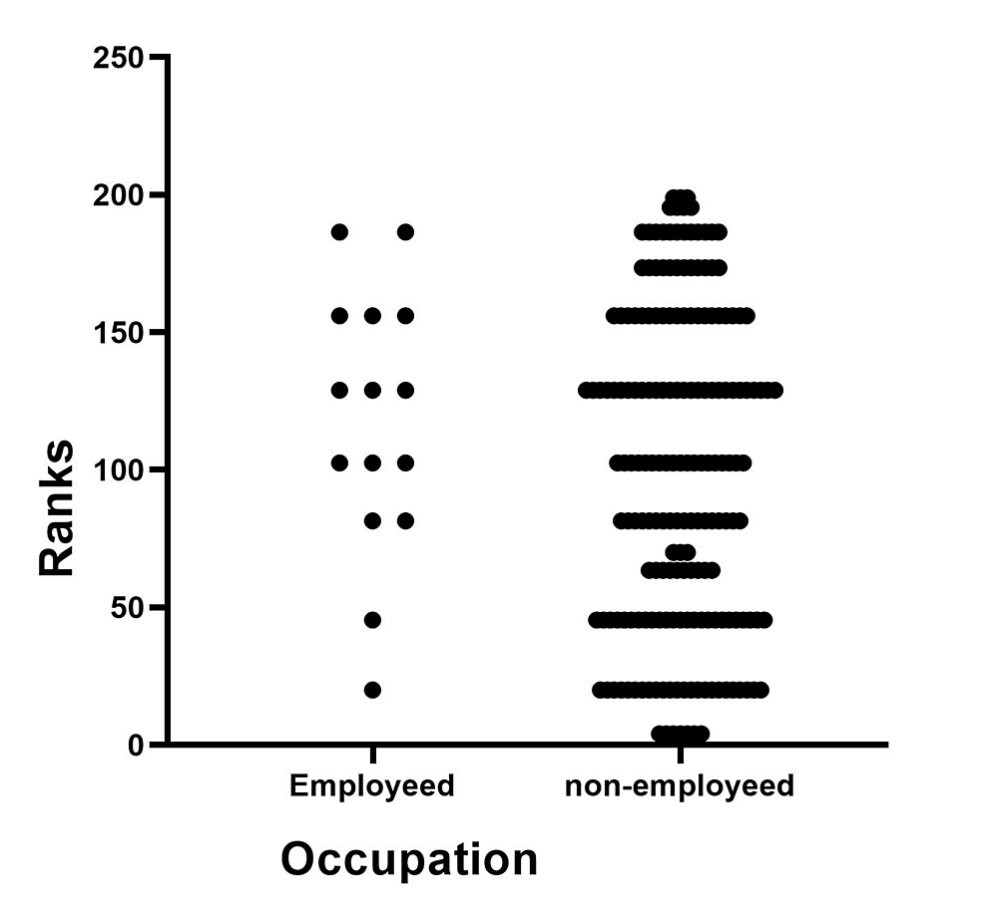
Difference between occupation and knowledge score. The Mann-Whitney U test was used. p≤0.05

The results of the Mann-Whitney U test showed that there were no significant differences in knowledge scores between patients diagnosed with PIH and PE. Likewise, no significant differences in knowledge scores were found based on age groups or occupation, as shown in Figures 2-4.

In relation to the results obtained from the Kruskal-Wallis test, no significant differences were found between the knowledge score and economic status, gravida, parity, and presence of previous abortion. However, there were a few significant differences observed with respect to the place of residence. Patients in rural areas exhibited higher knowledge scores compared to those from suburban areas (p value = 0.034). Furthermore, overweight patients displayed higher knowledge scores compared to obese pregnant women, and this relationship was highly significant (p value = 0.007). Regarding educational level, pregnant women who were illiterate had lower knowledge scores compared to those at the primary level and in institutes/colleges (p value = 0.002, 0.001, respectively) (Table 5).

**Table 5:** Association of knowledge score with different variables. vs. = Versus, BMI = Body mass index. The Kruskal-Wallis test was used. p≤0.05

Variables	Mean rank 1	Mean rank 2	Mean rank difference	p value
Economic status				
Low economic status vs. Enough for life	109.9	93.43	16.44	0.256
Low economic status vs. Good economic status	109.9	98.50	11.38	0.821
Enough for life vs. Good economic status	93.43	98.50	-5.066	>0.999
Resident place				
Urban vs. Rural	97.85	115.0	-17.15	0.197
Urban vs. Sub-urban	97.85	74.56	23.29	0.349
Rural vs. Sub-urban	115.0	74.56	40.44	0.034
BMI				
Normal BMI vs. Overweight	49.00	112.6	-63.65	0.176
Normal BMI vs. Obese	49.00	87.77	-38.77	0.752
Overweight vs. Obese	112.6	87.77	24.88	0.007
Gravida				
Primigravida vs. Multigravida	90.02	103.2	-13.15	0.578
Primigravida vs. Grand multigravida	90.02	104.4	-14.34	>0.999
Multigravida vs. Grand multigravida	103.2	104.4	-1.195	>0.999
Parity				
Nullipara vs. Primipara	94.10	37.00	57.10	0.585
Nullipara vs. Multipara	94.10	103.6	-9.507	>0.999
Nullipara vs. Grand mutlipara	94.10	99.33	-5.231	>0.999
Primipara vs. Multipara	37.00	103.6	-66.61	0.282
Primipara vs. Grand mutlipara	37.00	99.33	-62.33	0.559
Multipara vs. Grand mutlipara	103.6	99.33	4.276	>0.999
Abortion				
None vs. 1-2 times	106.2	95.66	10.57	0.611
None vs. 3-4 times	106.2	80.44	25.80	0.665
1-2 times vs. 3-4 times	95.66	80.44	15.23	>0.999
Education level				
Illiterate vs. Primary	65.07	106.9	-41.86	0.002
Illiterate vs. Secondary	65.07	96.7	-31.63	0.146
Illiterate vs. Institute and college	65.07	125.8	-60.78	0.001
Primary vs. Secondary	106.9	96.7	10.23	>0.999
Primary vs. Institute and college	106.9	125.8	-18.92	>0.999
Secondary vs. Institute and college	96.7	125.8	-29.14	0.428

The regression analysis revealed several significant predictors of the dependent variable. Education level showed a statistically significant positive association (β = 0.219; 95% CI 0.322 to 1.379; p = 0.002), indicating that higher education levels were associated with an increase in the outcome variable. However, place of residence demonstrated no significant association (β = -0.012; 95% CI -0.728 to 0.614; p = 0.866), suggesting that urban or rural residence did not significantly influence the outcome. Another significant predictor was BMI, which showed a negative association with the dependent variable (β = -0.185; 95% CI -1.934 to -0.293; p = 0.008). This finding implies that higher BMI was associated with a decrease in the outcome variable (Table 6).

**Table 6:** Linear regression result of knowledge score with the other factors. B = Unstandardized regression coefficient, CI = Confidence interval. The linear regression analysis test was used. p≤0.05

Model	B	Beta	p value	95% CI	
(Constant)	7.411		˂0.001	4.116	10.705
Education level	0.85	0.219	0.002	0.322	1.379
Place of residence	-0.057	-0.012	0.866	-0.728	0.614
Body Mass Index	-1.113	-0.185	0.008	-1.934	-0.293

## Discussion

This study reveals that 72% of the pregnant participants in the Kurdistan region had insufficient understanding of PE. Additionally, only 17.5% of individuals had moderate awareness, and a mere 10.5% had a high level of knowledge about PE. The finding that most participants' knowledge of PE primarily came from their understanding of chronic hypertension can be attributed to the population's limited familiarity with PE. Nonetheless, only a small percentage of participants were well-informed about the signs, causes, and consequences of PE. Firstly, the study found that level of education, place of residence, and BMI had an impact on the knowledge score. When using linear regression analysis, the results indicated that the model suggests education level positively influences the knowledge score, while BMI has a negative impact. Place of residence does not appear to have a significant effect on the knowledge score.

Previous studies have shown that women have a lack of knowledge about PE. In a study conducted in the United States by You et al., it was found that women had a knowledge level of only 43.3% about PE, and only 14% were able to correctly identify the illness [16]. Similarly, a study conducted in Malaysia by Zuo et al. revealed that only 18.4% of women had sufficient knowledge about PE [19]. In Ghana, research demonstrated that 88.6% of individuals had an inadequate understanding of PE, with a mean score of 55.5 ± 4.3% [18]. On the other hand, 11.4% of participants displayed adequate knowledge, with a mean score of 76.3 ± 5.9%. Among those with adequate knowledge, 2.3% (mean score = 85.2% ± 5.1%) and 9.1% (mean score = 67.4 ± 6.9%) exhibited moderate and high levels of knowledge, respectively [18].

According to additional research conducted by Eze et al. [11] and Savage and Hoho [5], it was found that 59% and 60% of Tanzanian women, respectively, lacked sufficient knowledge about PE. This evidence suggests that a solid understanding of the disorder is crucial for its prevention, control, and management. When patients are aware of their condition, they are more likely to comply with therapy and experience reduced difficulties associated with it [20].

This finding implies a correlation between improved clinical outcomes and a comprehensive understanding of PE, as well as vice versa. To address the increasing incidence of the disease and its adverse consequences, it may be essential to evaluate and enhance the awareness of high-risk individuals, particularly pregnant women, regarding PE. When women are informed about the potential implications of the symptoms they experience, they are more inclined to seek prompt medical attention. Although the low level of PE knowledge identified in this study is concerning, it could potentially be improved as the factors influencing PE knowledge were not static or broadly based on demographic factors. After controlling for variables that could skew the correlation, it was apparent that a high level of education was the only significant factor independently associated with adequate knowledge of PE. Hence, this research suggests that implementing an effective education method for women, possibly during prenatal visits and through media channels, could enhance their understanding of PE and significantly contribute to the ongoing efforts in Ghana and across Africa to reduce the mortality rate associated with PE. Studies have shown that increasing patient awareness of PE can promote early reporting of symptoms, leading to prompt treatment and better outcomes for both the mother and child. Moreover, a study conducted in Ethiopia reported slightly better results than the recent study, revealing that 28.8% of Ethiopian women had good knowledge about PE [15].

The findings of our investigation revealed a significant association between the educational attainment of the study participants and their level of knowledge regarding PE. Individuals who had completed basic school education or higher demonstrated a greater likelihood of possessing knowledge about PE, in comparison to those who had not received any formal education. This finding is consistent with previous studies conducted in India [21] and Ghana [18]. Another study conducted in Saudi Arabia reported that 39.3% of respondents had some level of awareness regarding PE, while only 11.3% possessed sufficient knowledge, and 49.4% had no knowledge at all. Notably, a higher level of knowledge was strongly associated with advanced age, previous experience with pregnancy, and a family history of PE [22].

PE may not be well-known due to a lack of personal experience, suitable health companions, and social media coverage. However, this study demonstrates that this issue is not limited to specific regions but is a global problem. Researchers and policymakers seeking to raise awareness about PE on a larger scale can benefit from this perspective. It is essential to acknowledge the limitations of this study. Firstly, the sample used does not accurately represent the overall population of Kurdistan. Additionally, since women self-reported the data used in the study, there may be biases related to social desirability or recall that could have influenced the findings. The results of the study may be skewed due to potential under- or overreporting of PE awareness by some individuals. It is important to note that the study's questionnaire only recorded participants' awareness and knowledge at a specific moment in time, without considering any advancements or changes over time. Cross-sectional studies are prone to confounding variables that may impact both the exposure and the outcome. Despite statistical techniques being able to address certain confounders, it remains challenging to consider all potential confounding factors. Future research should prioritize improving sample representation, addressing biases in self-reported data, conducting longitudinal studies, and exploring cultural and socioeconomic influences on awareness.

## Conclusions

The present study offers a comprehensive evaluation of the understanding of preeclampsia (PE) among Kurdish women, emphasizing areas where intervention and educational programs could be beneficial. By pinpointing knowledge gaps, particularly among younger women, specific awareness campaigns can be designed to tackle this issue. In addition, it is crucial for community midwives and nurses to conduct home visits for pregnant women, utilizing digital devices to record vital signs and collect urine samples. This strategy plays a pivotal role in the prompt detection of PE. Furthermore, our research suggests preventive measures that could potentially decrease the incidence of PE, ultimately improving maternal health. This study has the potential to inform healthcare policies and initiatives not only in Kurdistan but also in other settings, thus resulting in better health outcomes for mothers.

## Appendices

Appendix A

**Table 7:** Knowledge about PIH and preeclampsia. HDP: Hypertensive disorders in pregnancy, PIH: Pregnancy-induced hypertension.

No.	Knowledge	Yes	No
1	Have you heard about HDP/preeclampsia?		
2	PIH and preeclampsia become hypertension after 20 weeks?		
3	Do you know the signs and symptoms of PIH and preeclampsia?		
4	Is the family history of hypertension a risk factor for HDP/preeclampsia?		
5	Is pre-pregnancy obesity a risk factor for PIH and preeclampsia?		
6	Are PIH and preeclampsia in previous pregnancy a risk factor for PIH and preeclampsia in current pregnancy?		
7	Is rapid weight gain in pregnancy a risk factor for HDP/preeclampsia in pregnancy?		
8	Is measuring urine tests necessary in pregnancy?		
9	Is measuring blood pressure necessary in pregnancy?		
10	Can diet treat PIH and preeclampsia?		
11	Can exercise treat PIH and preeclampsia?		
12	Can a baby be harmed if PIH and preeclampsia are not treated?		

Appendix B 

**Table 8:** Sociodemographic, obstetric, and clinical parameters. PIH = Pregnancy-induced hypertension.

Medical diagnosis
-Preeclampsia
-PIH
Maternal age (years)
Education level
-Illiterate
-Primary
-Secondary
-Institute/university
-Postgraduate
Occupation
-Employed
-Non-employed
Financial status
-Low income
-Enough for life
-Good economic status
Place of residence
-Urban
-Rural
-Sub-rural
BMI (kg/m2)
Obstetric history
-Gravida
-Parity
-Abortion
-Dead
Blood pressure
-Systolic blood pressure
-Diastolic blood pressure
Hemoglobin level (g/dl)
Urine test for albumin
